# The recent ancestry of Middle East respiratory syndrome coronavirus in Korea has been shaped by recombination

**DOI:** 10.1038/srep18825

**Published:** 2016-01-06

**Authors:** Jin Il Kim, You-Jin Kim, Philippe Lemey, Ilseob Lee, Sehee Park, Joon-Yong Bae, Donghwan Kim, Hyejin Kim, Seok-Il Jang, Jeong-Sun Yang, Hak Kim, Dae-Won Kim, Jeong-Gu Nam, Sung Soon Kim, Kisoon Kim, Jae Myun Lee, Man Ki Song, Daesub Song, Jun Chang, Kee-Jong Hong, Yong-Soo Bae, Jin-Won Song, Joo-Shil Lee, Man-Seong Park

**Affiliations:** 1 Department of Microbiology, the Institute for Viral Diseases and Korea Bank for Pathogenic Viruses, College of Medicine, Korea University, Seoul 136-705, Republic of Korea; 2 Division of Respiratory Viruses; 3 Division of Influenza Virus, Center for Infectious Diseases; 4 Division of Biosafety Evaluation and Control; 5 Korea National Institute of Health, Osong 361-951, Republic of Korea; 6 Department of Microbiology and Immunology, Yonsei University College of Medicine, Seoul 120-752, Republic of Korea; 7 Laboratory Science Division, International Vaccine Institute, Seoul 151-742, Republic of Korea; 8 College of Pharmacy, Korea University, Sejong 339-700, Republic of Korea; 9 Division of Life & Pharmaceutical Sciences, Ewha Womans University, Seoul 120-750, Republic of Korea; 10 ATGen, Seongnam 463-400, Republic of Korea; 11 Department of Biological Science, Sungkyunkwan University, Suwon 440-746, Republic of Korea; 12 The MERS Task Force of the Korean Society of Virology, Seoul 137-701, Republic of Korea; 13 Department of Microbiology and Immunology, Rega Institute, KU Leuven – University of Leuven, Leuven 3000, Belgium

## Abstract

Middle East respiratory syndrome coronavirus (MERS-CoV) causes severe cases of human respiratory disease. Since 2012, the victims have mainly come from the Middle East countries or sporadically from some other geographical regions seeded by the travelers who visited the Middle East. Such an introduction through travelling led to the emergence of a MERS-CoV outbreak in Korea in May 2015, which caused more than 140 confirmed human cases in less than a month. Using 70 complete genome sequences of MERS-CoV isolates, including the most recent sequences for the Korean and Chinese isolates, we reconstructed the phylogenetic relationships of the complete genome and the individual protein coding regions. The Korean MERS-CoV strain clustered in the previously established Hafr-Al-Batin-1_2013 clade together with two Saudi Arabian and one Chinese strain sampled in 2015. Although these four strains remained monophyletic in the entire protein-coding region, this clade showed different phylogenetic relationships across the genome, indicating a shared unique recombination pattern that is different from previously reported putative recombination strains. Our findings suggest that the recent ancestor of the Korean and its related MERS-CoV strains is characterized by unique mosaic genome pattern that is different from other putative recombinants.

MERS was first described in 2012 in fatal human cases that were caused by a single-stranded RNA coronavirus^1^,^2^,^3^. Since then, more than 1,000 cases of MERS-CoV infection have been confirmed with an estimated case-fatality rate (CFR) of 39.5%^4^, and the elderly and immunocompromised patients appear to be most severely affected^5^. Acute respiratory distress and pneumonia are the major clinical manifestations of MERS-CoV infection^6^. Gastrointestinal symptoms and renal failure are also reported in some cases^5^,^7^. Dromedary camels are considered to be the intermediate host in MERS zoonotic transmission chains from bats to humans^8^,^9^. In a previously published study, the spread of MERS-CoV appeared to match transmission routes between animal reservoirs and infected humans^10^. However, it was not readily replicated in a camel transmission study^11^. Despite this contradiction, MERS-CoV spread was demonstrated largely among camels and people in the Middle East^12^ and travellers who visited the region could occasionally seed the virus to long-distance destinations in the Europe, Southeast Asia, and North America^9^,^13^,^14^.

The first case of traveler-associated MERS-CoV outbreak in Korea occurred in May 2015^15^. A 68-year-old index person traveled to four countries in the Middle East and returned to Korea on May 4 without clinical complaints. When clinical symptoms were developed one week later, the index person sought medical attention in two primary clinics and two upper-class hospitals, but a diagnosis was only made on May 20th by confirming MERS-CoV infection. In the meantime, one of the nosocomial contacts of the index patient travelled to China via Hong Kong and was diagnosed with MERS-CoV infection on May 27 in Guangdong. As of June 19, 2015, a total of 166 confirmed cases have been reported in the MERS outbreak in Korea including one Chinese case^16^. Even though the viral spread is mainly limited to hospital-based transmission, as seen in previous cases^17^,^18^ and no more confirmed cases are reported in Korea, this represents the largest outbreak outside the Middle East region. To investigate the evolutionary history of the MERS-CoV strain (GenBank accession No. KT029139; KOR-KNIH-002_2015, KOR002) responsible for the outbreak in Korea, we analyzed 70 complete genome sequences available in the NCBI including the most recent Chinese (KT006149; China-GD01_2015, China01) and Saudi Arabian (KT026455; Riyadh-KSA-2959_2015, KSA2959 and KT026456; Riyadh-KSA-4050_2015, KSA4050) sequences (Table S1).

We first analyzed the phylogenetic relationships between the KOR002 strain and all available MERS-CoV sequences. The complete genome and individual open reading frame (ORF) sequences (ORF1ab, S, ORF3, ORF4a, ORF4b, ORF5, E, N, M, and 8b) were analyzed separately using time-resolved Bayesian phylogenetic inference method implemented in BEAST (v1.8.2)^19^. In agreement with previous analyses^20^, six distinct clades were identified in the complete genome tree (Fig. 1a): Clade A, Riyadh-3, Jeddah-Riyadh, Hafr-Al-Batin-1, Buraidah-1, and Al-Hasa. Together with the Chinese China01 strain, the KOR002 strain clusters within the Hafr-Al-Batin-1 clade and shows a relatively close relationship with two Saudi-Arabian strains sampled in 2015 (KSA2959 and KSA4050). Other 2015 strains from Riyadh in Saudi Arabia (KR011264, Riyadh-2343_2015; KR001265, Riyadh02466_2015; KR011266, Riyadh-2049_2015; and KR011263, Riyadh-2345_2015) clustered in a different clade (Fig. 1a). Among the ten individual ORF trees, the tree of ORF1ab exhibited a similar pattern to that of complete genome sequences except for Bisha-1_2012 (GenBank accession number KF600620), Riyadh-1_2012 (KF600612), and Munich-UAE_2013 (KF192507) strains, which clustered together with other strains of the Riyadh-3 clade (Fig. 1b). In the complete genome tree, they were found basal to the Al-Hasa (Bisha-1_2012 and Riyadh-1_2012) and Riyadh-3 (Munich-UAE_2013) clades, respectively (Fig. 1a). Based on the phylogenetic trees of the complete genome and ORF1ab, the Hafr-Al-Batin-1 clade appeared to be more closely related to the Al-Hasa and Buraidah-1 clades whereas the Riyadh-3 and Jeddah-Riyadh clades formed a sister lineages (Fig. 1). In the trees of the S, ORF3, ORF4a, ORF4b, ORF5, E, and M genes, however, the most recent four sequences (KOR002, China01, KSA2959, and KSA4050) from the complete genome Hafr-Al-Batin-1 clade exhibited a closer relationship to the Jeddah-Riyadh strains compared to the other Hafr-Al-Batin-1, Al-Hasa, and Buraidah-1 clade sequences (Fig. 2a and S1B, S2, S3, and S4A). For the N and ORF8b genes, similar relationships were observed only among the Riyadh 2015 strains from the Jeddah-Riyadh clade sequences (Fig. 2b and S4B). This incongruent clustering pattern in the phylogenetic trees suggests that genetic recombination occurred in the MERS-CoV evolutionary history.

Recombination has been described previously in other coronavirus genomes^21^,^22^,^23^, and was also suggested to affect the evolution of MERS-CoV^24^. In our recombination analyses of the MERS-CoV complete genomes, 25 strains (2 strains in 2012, 10 strains in 2013, 9 in 2014, and 4 in 2015) emerged as putative recombinants (Table 1). The 20 strains isolated before 2015 appeared to retain two recombination breakpoints in the linear ORF alignment (Fig. 3a). However, four putative recombinants in 2015 (KOR002, China01, KSA2959, and KSA4050), coinciding with the strains showing the unique relationships noted in the phylogenetic trees above (Figs 1 and 2 and S1 to S4), shared four breakpoints, which resulted in five recombinant fragments (Fig. 3b). Based on these mosaic patterns shared among the four putative recombinants in 2015, we compiled five new datasets representing each non-recombinant fragment and evaluated the phylogenetic relationships of the four putative recombinants in 2015. In each tree (Fig. 3c–e and S5), the four putative recombinants in 2015 always grouped together and showed close relationships with their parental strains as detected in the recombination test (Table 1). A recombination analysis using a larger window size suggested similar strains as putative recombinants, especially for KOR002 and its related 2015 strains (Table S2). Consistent with phylogenetic results above, the trees of each recombination region exhibited similar evolutionary clustering patterns according to the inclusion of corresponding ORF regions: in the trees of recombination region II and IV (Fig. 3d and S1B, S2, S3, and S4A), which represent the ORF1ab and a large part of the S-M protein coding regions, each clade clustered similar to the trees in Fig. 1b and S1A, respectively. In the recombination region III (18,033 to 23,502 region; 5,470 nucleotides in length) (Fig. 3b), which comprises the region of C-terminal ORF1ab and N-terminal S protein genes, the tree pattern appeared to be similar to that of S protein gene (Figs 2a and 3e), which is characterized by a much higher substitution rate than the ORF1ab (Table S3). Even though we used cell culture media of the third passaged Vero cells for the RNA isolation of the KOR002 strain, the possibility for contamination of the original sputum sample with multiple viral clones and subsequent recombination can be excluded because the Chinese and Saudi Arabian strains related to KOR002 all exhibited similar genomic recombination patterns. Taken together, these results suggest that genetic recombination has contributed to the evolutionary dynamics of MERS-CoV genomes and that this has particularly shaped the recent MERS-CoV ancestry of the Korean outbreak.

Based on the phylogenetic clustering patterns and the recombination imprints we detected (Figs 1, 2, 3 and Table 1), one of the recombinant strains that evolved from the Hafr-Al-Batin-1 clade was introduced by air travel into Korea. We can only speculate about when the genetic recombination occurred. However, in the Riyadh area, some strains of the Jeddah-Riyadh clade already circulated before May 2015, and considering the close relationships between some of the Hafr-Al-Batin-1 and Jeddah-Riyadh clade sequences shown in the phylogenetic trees, especially in Fig. 3e, genetic exchange appeared to have occurred among them and affected the phylogenetic evolution of MERS-CoV lineages before the Korean traveler was infected by a productive recombinant strain in the area^24^. However, as discussed previously with regard to the emergence of severe acute respiratory syndrome coronavirus (SARS-CoV) in 2002, other evolutionary aspects, such as mutation rates and selection pressure, should be considered to understand the evolutionary dynamics of MERS-CoV^21^,^25^,^26^. Possibly different molecular clock rates of MERS-CoV in animal hosts and humans may also have to be taken into account. As shown by the genomic evolution of influenza A viruses^27^, MERS-CoV might experience different evolutionary courses in different hosts. To better understand these dynamics, the chain of MERS-CoV zoonotic transmissions should be further clarified.

Outside the Arabian Peninsula, Korea experienced the biggest outbreak of MERS. Through seeding by only a single patient, MERS-CoV resulted in more than 160 confirmed patients in less than a month and thousands of people were confined under close monitoring. The CFR of MERS outbreak in Korea may appear to be relatively low (approximately 11.7%), compared with the previous outbreaks in the Middle East and no signs of community transmission have been reported. In addition, an announcement regarding the situation assessment of MERS outbreak in Korea issued by the WHO Global Alert and Response program stated that significant virological change was not seen so far and the transmission patterns are unlikely to be different from those previously reported in the Middle East^16^. However, human infections with the MERS-CoV are ongoing in the Middle East countries, and the virus may travel anywhere from the region as seen in the current Korean outbreak and many other previous cases. In support of the struggle against the relatively new MERS-CoV infection, effective medical arsenals should be prepared using the comprehensive measures of epidemiology, pathogenesis, and transmission researches.

In conclusion, we suggest that the MERS-CoV outbreak in Korea appears to be caused by a strain that is closely related to three 2015 strains from the Hafr-Al-Batin-1 clade and that the relatively recent ancestor of these viruses exhibits a unique recombination pattern that is different from other putative recombinants.

## Methods

### Sequence preparation

In this study, we investigated a total of 70 complete genome sequences of human MERS-CoV strains that were downloaded from the database of National Center for Biotechnology Information (NCBI; http://www.ncbi.nlm.nih.gov/genomes/VirusVariation/Database/nph-select2.cgi). Detailed information of isolation and genomic sequencing of a Korean MERS-CoV strain was published previously^28^. Briefly, a sputum sample was collected in May 20, 2015 from the wife of the index case, who travelled to four Middle East countries including the United Arab Emirates and the Kingdom of Saudi Arabia for 16 days before the onset of his symptom in May 11, 2015. After the third passage in Vero cells, viral RNA was obtained from cell culture media (QIAamp viral RNA mini kit: QIAGEN, Germany) and used for reverse-transcription PCR (Superscript III first-strand synthesis system: Life Technologies, the Netherlands)^29^. The pooled PCR amplicons were then fragmented (~ 300 bp in length) and used for the sequence library construction using an Illumina TruSeq Nano DNA sample prep kit (Illumina, San Diego, CA, USA). After sequencing the library using an Illumina MiSeq 50-bp single-end platform (Illumina), more than 2.6 million sequence reads out of 2,814,805 reads (approximately 93% usage) were mapped to the consensus of human-origin MERS-CoV genome sequences downloaded from GenBank using Bowtie version 2.2.4^30^. The complete genome sequence was obtained based on an average coverage of 3,605.95 and submitted to the NCBI database (accession number KT029139, MERS-CoV/KOR/KNIH/002_05_2015)^28^. The first Chinese strain of MERS-CoV (NCBI accession number KT006149, Middle East respiratory syndrome coronavirus strain ChinaGD01) appeared to be imported from a Korean traveler who visited to Guangdong Province and was sequenced directly from a nasopharyngeal sample (collected in May 27, 2015) using a NGS method^31^. The two most recent Saudi Arabian sequences in 2015 appeared to be obtained after passages in Vero cells of a tracheal aspirate (NCBI accession number KT026455, Hu/Riyadh_KSA_2959_2015)^32^ and a respiratory swab (NCBI accession number KT026456, Hu/Riyadh_KSA_4050_2015)^33^ samples using the NGS method. After alignment using the MAFFT program (v7.130b)^34^, the datasets of complete genome and 10 protein coding regions were established by extracting corresponding sequence regions. The stop codon in the C-terminal region was removed. The resulting MERS-CoV datasets were assigned to complete genome (29,529 nucleotides, nts), ORF1ab (21,234 nts)), S (4,059 nts), ORF3 (309 nts), ORF4a (327 nts), ORF4b (738 nts), ORF5 (672 nts), E (246 nts), M (657 nts), N (1,239 nts), and ORF8b (336 nts). To compare phylogenetic clustering patterns, we also set up another dataset for the regions S through N coding regions (S-N region; 8,352 nts).

### Phylogenetic trees and evolutionary dynamics

Phylogenetic relationships, evolutionary rates (nucleotide substitutions/site/year), and the time (year) of the most recent common ancestor (tMRCA) were estimated using a time-framed Bayesian evolution analysis approach via a Markov Chain Monte Carlo (MCMC) inference method, implemented in the BEAST package (v1.8.2)^19^. We used the GTR+I+Γ substitution model, a lognormal relaxed molecular clock model and a Bayesian skygrid tree prior. For the of ORF4a, ORF4b, and ORF5 datasets, we used the HKY+Γ substitution model and a strict clock model. The evolutionary parameters (only for substitution and molecular clock parameters, not the tree model) were linked for the dataset of E coding region by adjoining those of the complete genome sequences. MCMC analyses were run for 50 million iterations, sampling every 25 thousand iterations after a 10% burn-in. Two or three independent runs for each dataset were combined and assessed to ensure their convergence in Tracer (v1.6)^35^. The MCMC tree samples were used to summarize a maximum clade credibility (MCC) trees for each dataset using TreeAnnotator v1.8.1, which were visualized using FigTree (v1.4.2). The estimates were presented as mean values along with the lower and upper limits of the 95% highest probability density (HPD).

### Recombination analysis

To detect putative recombinant regions in the MERS-CoV genome, we used the RDP4 program (v.4.39)^36^ with a default (window size: 30 bp) and a higher window size of 1,000 bp, and the results obtained were confirmed by a manual bootscan method. Using the recombination breakpoints detected in the KOR002 strain by the default setting, we compiled new sequence datasets by dividing the complete genome sequences into five non-recombinant fragments. We subsequently reconstructed the phylogenetic relationships in each region using a maximum likelihood method (GTR+I+Γ, 500 bootstrap replication) implemented in MEGA5^37^. The trees were visualized using FigTree (v1.4.2).

## Additional Information

**How to cite this article**: Kim, J. I. *et al.* The recent ancestry of Middle East respiratory syndrome coronavirus in Korea has been shaped by recombination. *Sci. Rep.* **6**, 18825; doi: 10.1038/srep18825 (2016).

## Supplementary Material

Supplementary Information

## Figures and Tables

**Figure 1:**
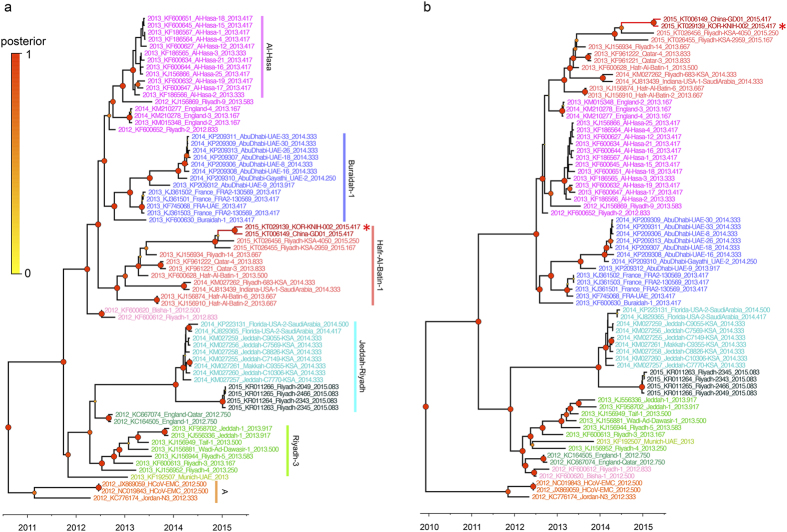
Phylogenetic relationships of MERS-CoV complete genome and ORF1ab sequences. The complete genome (**a**) and ORF1ab (**b**) sequences of 70 MERS-CoV strains were investigated for their phylogenetic relationships. In the complete genome tree, six different evolutionary clades were indicated with different colors (clade A, orange; Riyadh-3, lime green; Jeddah-Riyadh, mint; Hafr-Al-Batin-1, peach; Buraidah-1, lavender; and Al-Hasa, magenta). As the color of circles in the tree nodes, the size of circles in the node represents the posterior probability of their clustering (the bigger size, the higher probability). The tip of the Korean strain (KOR002) was denoted with the red color and asterisk.

**Figure 2:**
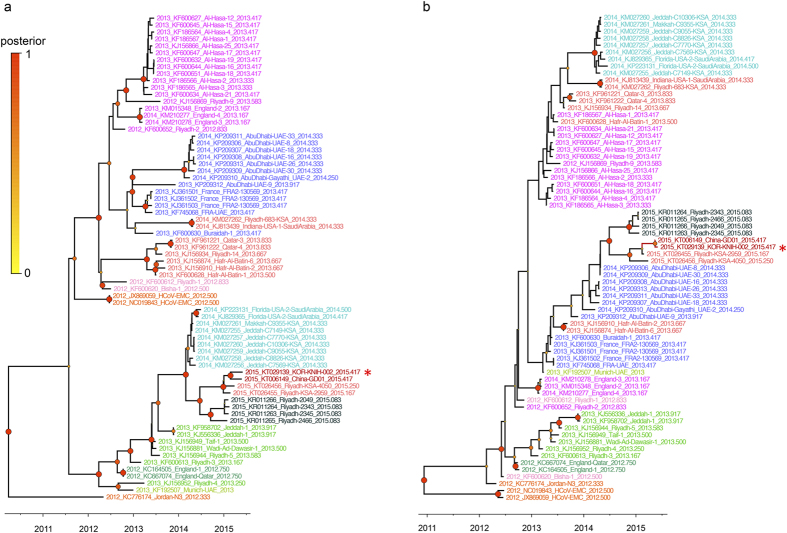
Phylogenetic relationships of MERS-CoV S and N sequences. The S (**a**) and N (**b**) sequences of 70 MERS-CoV strains were investigated for their phylogenetic relationships. See the detailed legend in the Fig. 1.

**Figure 3:**
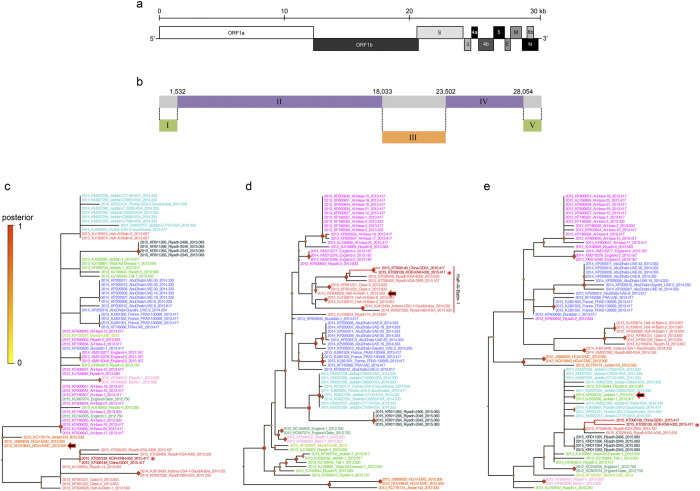
Schematic representation of MERS-CoV coding regions and putative recombinant regions detected in the KOR002 strain. The protein coding regions of MERS-CoV genome (**a**) were represented together with the putative recombinant regions of KOR002 strain (**b**) detected by the RDP method^36^ using the complete genome sequences of 70 MERS-CoV strains. Phylogenetic relationships of MERS-CoV complete genomes were reconstructed according to each recombinant region. The trees of recombinant regions I (**c**), II (**d**), and III (**e**) were represented with the same color annotations in the Fig. 1. The tip of the Korean strain (KOR002) was denoted with the red color and asterisk. The tip of a parental strain of KOR002 identified (Table 1) was indicated with an arrow.

**Table 1:** Putative recombinant strains (n = 25) detected by the RDP4 method with a default setting (window size = 30).

Accession #	Strain	Recombination region (nucleotide)	Corresponding ORF region	Parental strain	% similarity
KC164505	England-1_2012	22965–24618	S	KM027262_Riyadh-683-KSA_2014	99.5
KC667074	Enland-Qatar_2012	22602–24618	S	KF600620_Bisha-1_2012	99.5
KF600613	Riyadh-3_2013	22965–25594	S	KM027262_Riyadh-683-KSA_2014	99.4
KF600628	Hafr-Al-Batin-1_2013	231–1532	ORF1a	NC019843_HCoV-EMC_2012	99.8
KF958702	Jeddah-1_2013	22965–26708	S, ORF3, ORF4a, ORF4b, ORF5	KM027262_Riyadh-683-KSA_2014	99.5
KF961221	Qatar-3_2013	1–1532; 28497–29529	ORF1a, N, ORF8b	NC019843_HCoV-EMC_2012	99.8
KF961222	Qatar-4_2013	231–1532	ORF1a	NC019843_HCoV-EMC_2012	99.9
KJ156881	Wadi-Ad-Dawasir-1_2013	22965–25594	S	KM027262_Riyadh-683-KSA_2014	99.4
KJ156934	Riyadh-14_2013	231–1532	ORF1a	NC019843_HCoV-EMC_2012	99.9
KJ156944	Riyadh-5_2013	22965–26708	S, ORF3, ORF4a, ORF4b, ORF5	KM027262_Riyadh-683-KSA_2014	99.5
KJ156949	Taif-1_2013	22965–25594	S	KM027262_Riyadh-683-KSA_2014	99.5
KJ556336	Jeddah-1_2013	22963–26706	S, ORF3, ORF4a, ORF4b, ORF5	KM027262_Riyadh-683-KSA_2014	99.5
KJ829365	Florida-USA-2-Saudi Arabia_2014	22964–24397	S	KM027262_Riyadh-683-KSA_2014	99.3
KM027255	Jeddah-C7149-KSA_2014	22965–24397	S	KM027262_Riyadh-683-KSA_2014	99.4
KM027256	Jeddah-C7569-KSA_2014	22965–24397	S	KM027262_Riyadh-683-KSA_2014	99.4
KM027257	Jeddah-C7770-KSA_2014	22965–28690	S, ORF3, ORF4a, ORF4b, ORF5, E, M, N, ORF8b	KM027262_Riyadh-683-KSA_2014	99.7
KM027258	Jeddah-C8826-KSA_2014	22759–24397	S	KM027262_Riyadh-683-KSA_2014	99.4
KM027259	Jeddah-C9055-KSA_2014	22965–24397	S	KM027262_Riyadh-683-KSA_2014	99.4
KM027260	Jeddah-C10306-KSA_2014	22965–24397	S	KM027262_Riyadh-683-KSA_2014	99.4
KM027261	Makkah-C9355-KSA_2014	22965–24397	S	KM027262_Riyadh-683-KSA_2014	99.4
KP223131	Florida-USA-2-Saudi Arabia_2014	22964–24397	S	KM027262_Riyadh-683-KSA_2014	99.3
KT006149	China-GD01_2015	1–1532; 28054–29529	ORF1a, M, N, ORF8b	NC019843_HCoV-EMC_2012	99.8
		18033–23502	ORF1b, S	KF958702_Jeddah-1_2013	99.9
KT026455	Riyadh-KSA-2959_2015	1–1532; 28497–29529	ORF1a, N, ORF8b	NC019843_HCoV-EMC_2012	99.8
		17424–23502	ORF1b, S	KF958702_Jeddah-1_2013	99.9
KT026456	Riyadh-KSA-4050_2015	1–1532; 28497–29529	ORF1a, N, ORF8b	NC019843_HCoV-EMC_2012	99.7
		18033–23502	ORF1b, S	KF958702_Jeddah-1_2013	99.9
KT029139	KOR-KNIH-002_2015	1–1532; 28055–29520	ORF1a, M, N, ORF8b	NC019843_HCoV-EMC_2012	99.8
		18033–23502	ORF1b, S	KF958702_Jeddah-1_2013	99.9
