# Supplementary material for: The recent ancestry of Middle East respiratory syndrome coronavirus in Korea has been shaped by recombination
Source: Sci Rep. 2016 Jan 6;6:18825. doi: 10.1038/srep18825 (PMC4702133; doi:10.1038/srep18825)

Supplementary Information

The recent ancestry of Middle East respiratory syndrome coronavirus in Korea has been shaped by recombination.

Authors: Jin Il Kim, You-Jin Kim, Philippe Lemey, Ilseob Lee, Sehee Park, Joon-Yong Bae, Donghwan Kim, Hyejin Kim, Seok-Il Jang, Jeong-Sun Yang, Hak Kim, Dae-Won Kim, Jeong-Gu Nam, Sung Soon Kim, Kisoon Kim, Jae Myun Lee, Man Ki Song, Daesub Song, Jun Chang, Kee-Jong Hong, Yong-Soo Bae, Jin-Won Song, Joo-Shil Lee, Man-Seong Park

Table S1 Page 2
Table S2 Page 5
Table S3 Page 8

Figure S1 Page 9
Figure S2 Page 10
Figure S3 Page 11
Figure S4 Page 12
Figure S5 Page 13

Table S1. Information of complete genome sequences used in the study.

Year	GenBank accession No.	Stain name
2012	JX869059	HCoV-EMC_2012
	KC164505	England-1_2012
	KC667074	England-Qatar_2012
	KC776174	Jordan-N3_2012
	KF600612	Riyadh-1_2012
	KF600620	Bisha-1_2012
	KF600652	Riyadh-2_2012
	KJ156869	Riyadh-9_2013
	NC019843	HCoV-EMC_2012
2013	KF186564	Al-Hasa-4_2013
	KF186565	Al-Hasa-3_2013
	KF186566	Al-Hasa-2_2013
	KF186567	Al-Hasa-1_2013
	KF192507	Munich-UAE_2013
	KF600613	Riyadh-3_2013
	KF600627	Al-Hasa-12_2013
	KF600628	Hafr-Al-Batin-1_2013
	KF600630	Buraidah-1_2013
	KF600632	Al-Hasa-19_2013
	KF600634	Al-Hasa-21_2013
	KF600644	Al-Hasa-16_2013
	KF600645	Al-Hasa-15_2013
	KF600647	Al-Hasa-17_2013
	KF600651	Al-Hasa-18_2013
	KF745068	France-UAE_2013
	KF958702	Jeddah-1_2013
	KF96122	Qatar-3_2013
	KF961222	Qatar-4_2013
	KJ156866	Al-Hasa-25_2013
	KJ156874	Hafr-Al-Batin-6_2013
	KJ156881	Wadi-Ad-Dawasir-1_2013
	KJ156910	Hafr-Al-Batin-2_2013

	KJ156934	Riyadh-14_2013
	KJ156944	Riyadh-5_2013
	KJ156949	Taif-1_2013
	KJ156952	Riyadh-4_2013
	KJ361501	France FRA2-130569_2013
	KJ361502	France FRA2-130569_2013
	KJ361503	France FRA2-130569_2013
	KJ556336	Jeddah-1_2013
	KM015348	England-2_2013
	KM210277	England-4_2013
	KM210278	England-3_2013
	KP209312	AbuDhabi-UAE-9_2013
2014	KJ813439	Indiana-USA-1-Saudi Arabia_2014
	KJ829365	Florida-USA-2-Saudi Arabia_2014
	KM027255	Jeddah-C7149-KSA_2014
	KM027256	Jeddah-C7569-KSA_2014
	KM027257	Jeddah-C7770-KSA_2014
	KM027258	Jeddah-C8826-KSA_2014
	KM027259	Jeddah-C9055-KSA_2014
	KM027260	Jeddah-C10306-KSA_2014
	KM027261	Makkah-C9355-KSA_2014
	KM027262	Riyadh-683-KSA_2014
	KP209306	AbuDhabi-UAE-8_2014
	KP209307	AbuDhabi-UAE-18_2014
	KP209308	AbuDhabi-UAE-16_2014
	KP209309	AbuDhabi-UAE-30_2014
	KP209310	AbuDhabi-Gayathi UAE-2_2014
	KP209311	AbuDhabi-UAE-33_2014
	KP209313	AbuDhabi-UAE-26_2014
	KP223131	Florida-USA-2-Saudi Arabia_2014
2015	KR011263	Riyadh-2345_2015
	KR011264	Riyadh-2343_2015
	KR011265	Riyadh-2466_2015
	KR011266	Riyadh-2049_2015
	KT006149	China-GD01_2015

KT026455	Riyadh-KSA-2959_2015
KT026456	Riyadh-KSA-4050_2015
KT029139	KOR-KNIH-002_2015

Table S2. Putative recombinant strains (n = 24) detected by the RDP4 method with an increased window size (to a maximum 1,000 size).

Accession #	Strain	Recombination region (nucleotide)	Corresponding ORF region	Parental strain§	% similarity
KC164505	England-1_2012	7996-25491	ORF1a, ORF1b, S	KF600647_Al-Hasa-17_2013	99.9
KC667074	Enland-Qatar_2012	7996-25491	ORF1a, ORF1b, S	KF600647_Al-Hasa-17_2013	99.9
KF600612	Riyadh-1_2012	9857-23152	ORF1a, ORF1b, S	KF600647_Al-Hasa-17_2013	99.9
KF600620	Bisha-1_2012	10042-23152	ORF1a, ORF1b, S	KF600647_Al-Hasa-17_2013	99.9
KF192507	Munich-UAE_2013	7996-26917	ORF1a, ORF1b, S, ORF3, ORF4a	KF600613_ Riyadh-3_2013	99.7
KJ156944	Riyadh-5_2013	16703-28923	ORF1b, S, ORF3, ORF4a, ORF4b, ORF5, E, M, N	KT006149_China-GD01_2015	99.7
KJ156952	Riyadh-4_2013	9857-23152	ORF1a, ORF1b, S	KF600647_Al-Hasa-17_2013	99.8
KJ829365	Florida-USA-2-Saudi Arabia_2014	657-16602	ORF1a, ORF1b	KJ156881_Wadi-Ad-Dawasir-1_2013	99.8
		16704-24170	ORF1b, S	KF600628_Hafr-Al-Batin-1_2013	99.8
KM027255	Jeddah-C7149-KSA_2014	183-16703	ORF1a, ORF1b	KJ156881_Wadi-Ad-Dawasir-1_2013	99.8
		16704-24170	ORF1b, S	KF600628_Hafr-Al-Batin-1_2013	99.8
KM027256	Jeddah-C7569-KSA_2014	657-16602	ORF1a, ORF1b	KJ156881_Wadi-Ad-Dawasir-1_2013	99.8
		16704-24170	ORF1b, S	KF600628_Hafr-Al-Batin-1_2013	99.8
KM027257	Jeddah-C7770-KSA_2014	3080-16703	ORF1a, ORF1b	KJ156881_Wadi-Ad-Dawasir-1_2013	99.8
		16704-24170	ORF1b, S	KF600628_Hafr-Al-Batin-1_2013	99.8

KM027258	Jeddah-C8826-KSA_2014	657-16602	ORF1a, ORF1b	KJ156881_Wadi-Ad-Dawasir-1_2013	99.8
		16704-24170	ORF1b, S	KF600628_Hafr-Al-Batin-1_2013	99.8
KM027259	Jeddah-C9055-KSA_2014	657-16602	ORF1a, ORF1b	KJ156881_Wadi-Ad-Dawasir-1_2013	99.8
		16704-24170	ORF1b, S	KF600628_Hafr-Al-Batin-1_2013	99.8
KM027260	Jeddah-C10306-KSA_2014	657-16602	ORF1a, ORF1b	KJ156881_Wadi-Ad-Dawasir-1_2013	99.8
		16704-24170	ORF1b, S	KF600628_Hafr-Al-Batin-1_2013	99.8
KM027261	Jeddah-C9355-KSA_2014	183-16703	ORF1a, ORF1b	KJ156881_Wadi-Ad-Dawasir-1_2013	99.8
		16704-24170	ORF1b, S	KF600628_Hafr-Al-Batin-1_2013	99.8
KP223131	Florida-USA-2-SaudiArabia_2014	657-16602	ORF1a, ORF1b	KJ156881_Wadi-Ad-Dawasir-1_2013	99.8
		16704-24170	ORF1b, S	KF600628_Hafr-Al-Batin-1_2013	99.8
KR011263	Riyadh-2345_2015	17219-28652	ORF1b, S, ORF3, ORF4a, ORF4b, ORF5, E, M, N, ORF8b	KT006149_China-GD01_2015	99.8
		17219-24170	ORF1b, S	KF600628_Hafr-Al-Batin-1_2013	99.7
KR011264	Riyadh-2343_2015	17219-28652	ORF1b, S, ORF3, ORF4a, ORF4b, ORF5, E, M, N, ORF8b	KT006149_China-GD01_2015	99.8
		17219-24170	ORF1b, S	KF600628_Hafr-Al-Batin-1_2013	99.7
KR011265	Riyadh-2466_2015	17219-28912	ORF1b, S, ORF3, ORF4a, ORF4b, ORF5, E, M, N, ORF8b	KT006149_China-GD01_2015	99.8
		17219-24170	ORF1b, S	KF600628_Hafr-Al-Batin-1_2013	99.7
KR011266	Riyadh-2049_2015	17219-28652	ORF1b, S, ORF3, ORF4a, ORF4b, ORF5, E, M, N, ORF8b	KT006149_China-GD01_2015	99.8
		17219-24170	ORF1b, S	KF600628_Hafr-Al-	99.7

				Batin-1_2013	
KT006149	China-GD01_2015	16703-23502	ORF1b, S	KF600628_Hafr-Al-Batin-1_2013	99.8
KT026455	Riyadh-KSA-2959_2015	17425-23502	ORF1b, S	KF600628_Hafr-Al-Batin-1_2013	99.9
KT026456	Riyadh-KSA-4050_2015	15974-23695	ORF1b, S	KF600628_Hafr-Al-Batin-1_2013	99.9
KT029139	KOR-KNIH-002_2015	16703-23502	ORF1b, S	KF600628_Hafr-Al-Batin-1_2013	99.9

[§] Major parental strains were primarily indicated except for the cases that minor parental strains were only presented with a relative statistical significance.

Table S3. Time of most recent common ancestor and evolutionary rates of MERS-CoV coding regions.

Genetic region	tMRCA[§]	Evolutionary rate (1x10^{-3} substitution/site/year)
Complete genome	5.80 (4.71-7.17)[¶]	0.74 (0.60-0.92)
ORF1ab	6.31 (4.54-8.33)	0.58 (0.44-0.72)
S	4.96 (4.07-5.94)	1.24 (0.81-1.63)
ORF3	5.01 (3.66-6.87)	4.71 (1.90-8.24)
ORF4a	7.64 (4.11-12.64)	1.26 (0.37-2.34)
ORF4b	6.05 (3.98-9.07)	1.27 (0.44-2.30)
ORF5	4.53 (3.69-5.72)	1.53 (0.72-2.52)
E	4.87 (3.69-6.46)	1.26 (0.93-1.68)
M	4.80 (3.60-6.47)	1.53 (0.52-2.65)
N	4.50 (3.65-5.64)	1.46 (0.56-2.33)
ORF8b	4.66 (3.60-6.84)	1.67 (0.31-3.28)

[§] tMRCA, time of most recent common ancestor. [¶] Lower and upper limits of 95% highest probability density (HPD) were indicated in parenthesis.

Figure S1. Phylogenetic relationships of MERS-CoV S-ORF3-ORF4a-ORF4b-ORF5-E-M-N (S-N) region and ORF3 sequences.

The S-N region (a) and ORF3 (b) sequences of 70 MERS-CoV strains were investigated for their phylogenetic relationships. As seen in the complete genome tree (Figure1), the same colors represent the same individual sequences (clade A, orange; Riyadh-3, lime green; Jeddah-Riyadh, mint; Hafr-Al-Batin-1, peach; Buraidah-1, lavender; and Al-Hasa, magenta). As the color of circles in the tree nodes, the size of circles in the node represents the posterior probability of their clustering (the bigger size, the higher probability). The tip of Korean strain (KOR002) was denoted with the red color and asterisk.

Supplementary figure 1

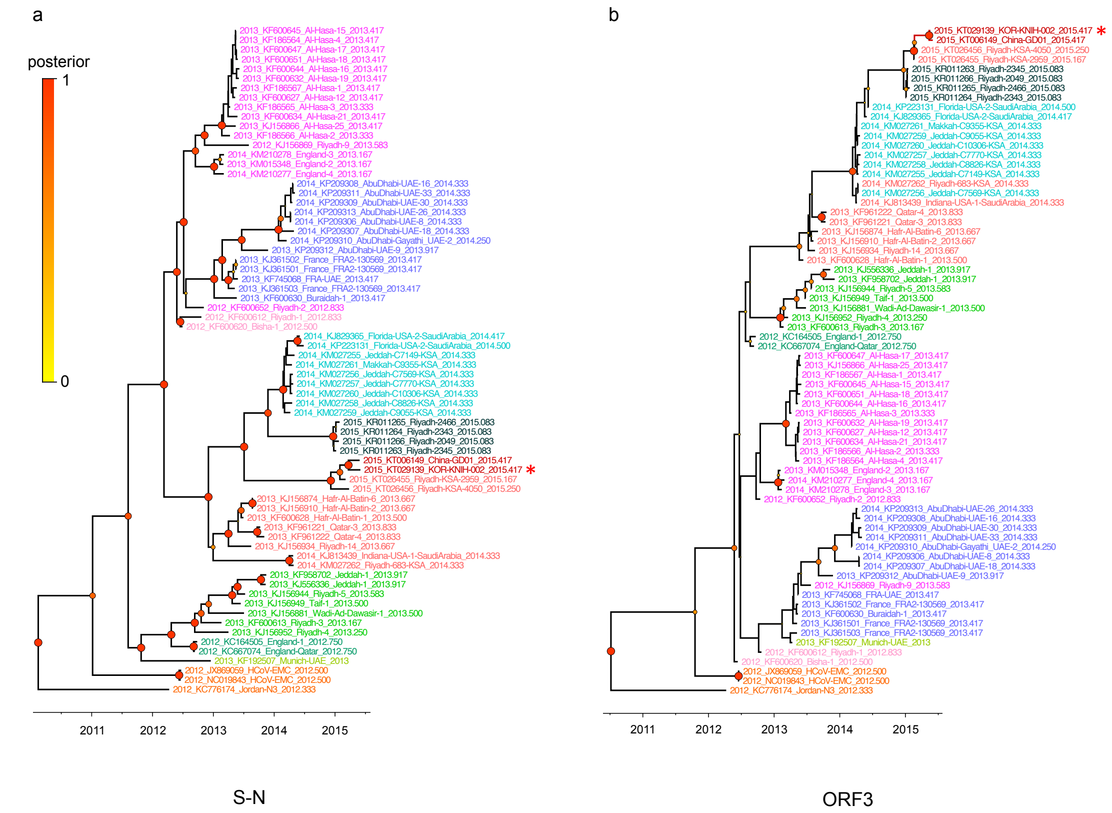

Figure S2. Phylogenetic relationships of MERS-CoV ORF4a and ORF4b sequences. The ORF4a (a) and ORF4b (b) sequences of 70 MERS-CoV strains were investigated for their phylogenetic relationships. For detailed information, please see the legend in the supplementary Figure 1.

Supplementary figure 2

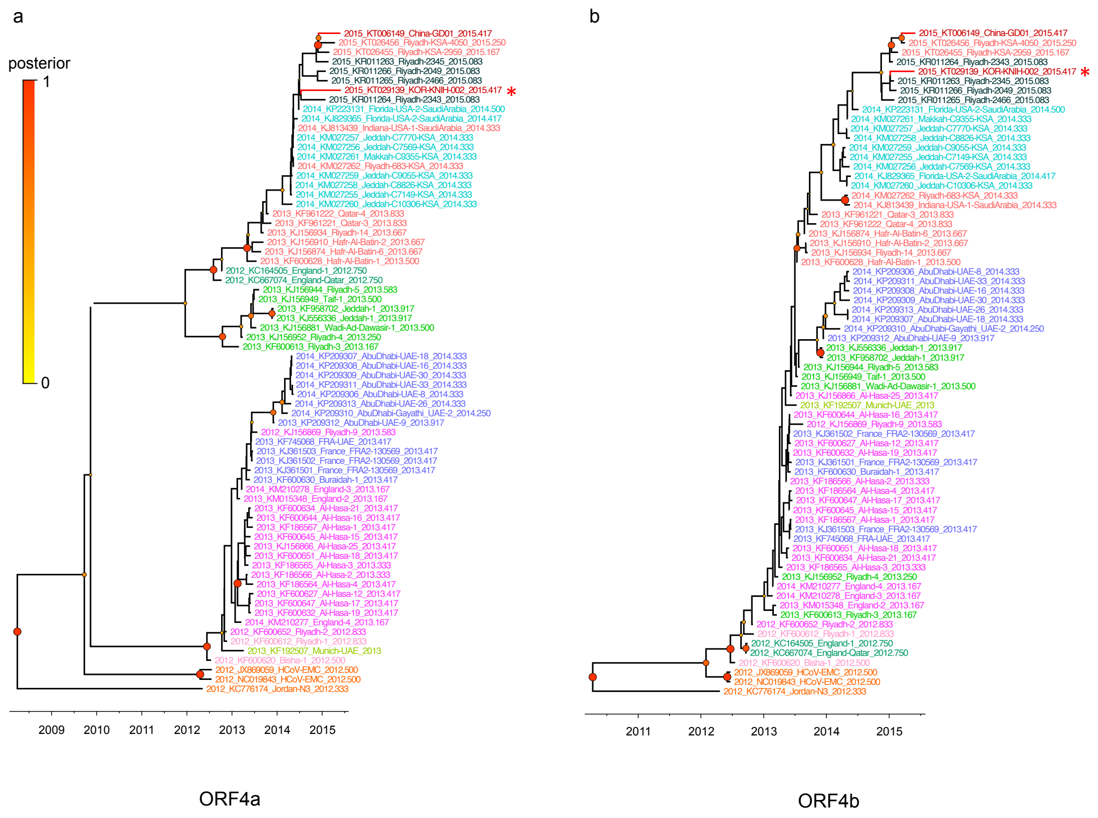

Figure S3. Phylogenetic relationships of MERS-CoV ORF5 and E sequences. The ORF5 (a) and E (b) sequences of 70 MERS-CoV strains were investigated for their phylogenetic relationships. For detailed information, please see the legend in the supplementary Figure 1.

Supplementary figure 3

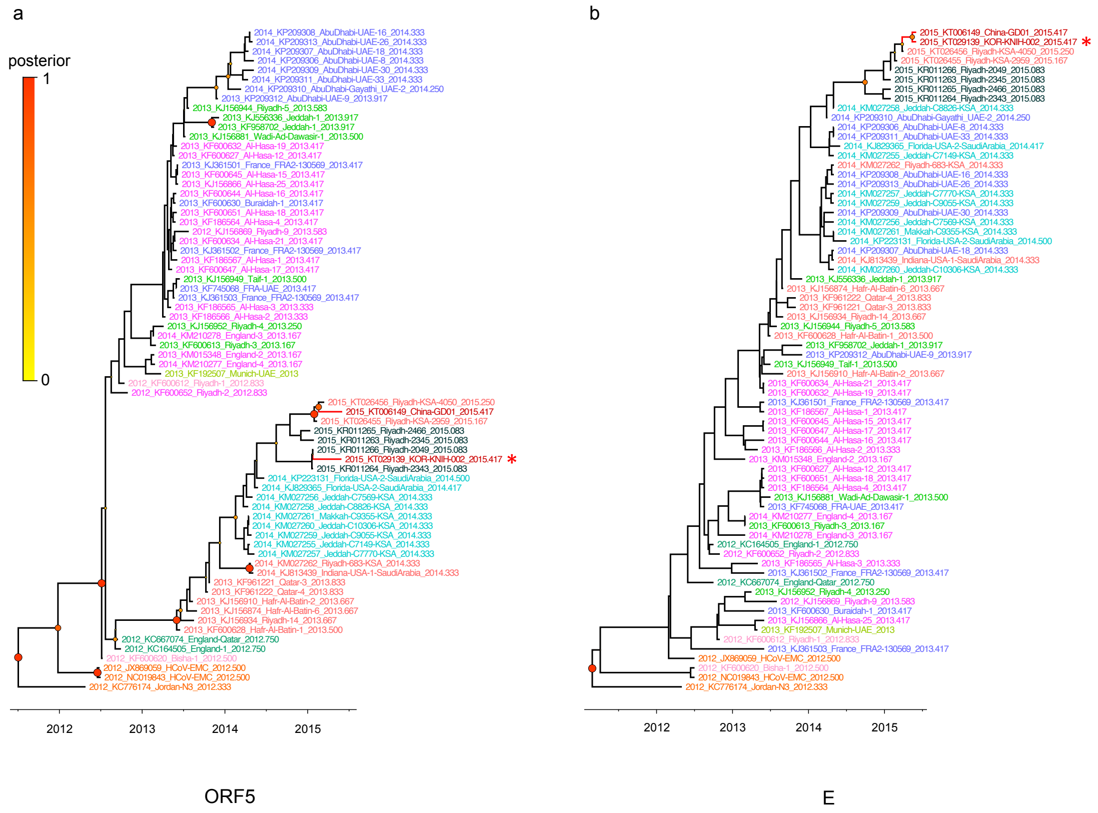

Figure S4. Phylogenetic relationships of MERS-CoV M and ORF8b sequences. The M (a) and ORF8b (b) sequences of 70 MERS-CoV strains were investigated for their phylogenetic relationships. For detailed information, please see the legend in the supplementary Figure 1.

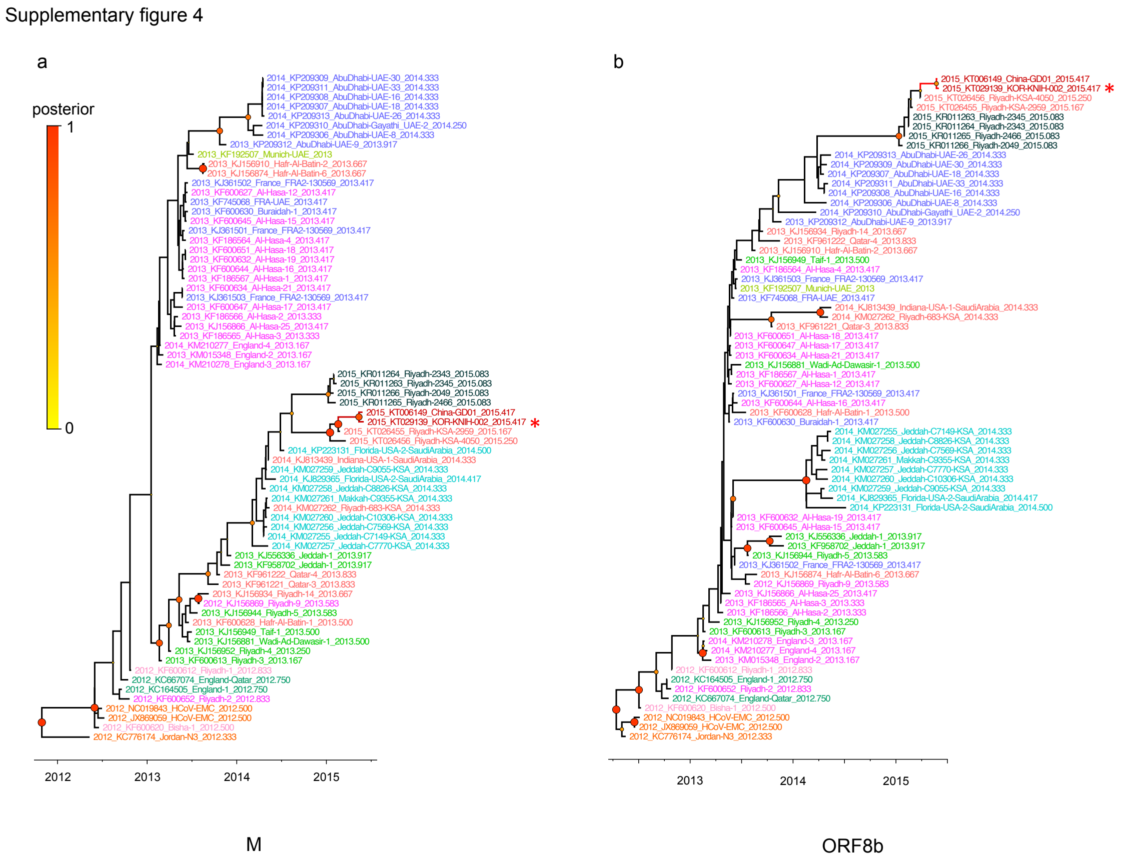

Figure S5. Phylogenetic relationships of putative recombinant regions detected in the KOR002 strain.

Phylogenetic relationships of MERS-CoV complete genomes were reconstructed according to each putative recombinant region detected in the KOR002 strain. The trees of recombinant regions IV (a) and V (b) were represented with the same color annotations in the Figure 1. The tip of the Korean strain (KOR002) was denoted with the red color and asterisk. The tip of a parental strain of KOR002 identified (Table 1) was indicated with an arrow.

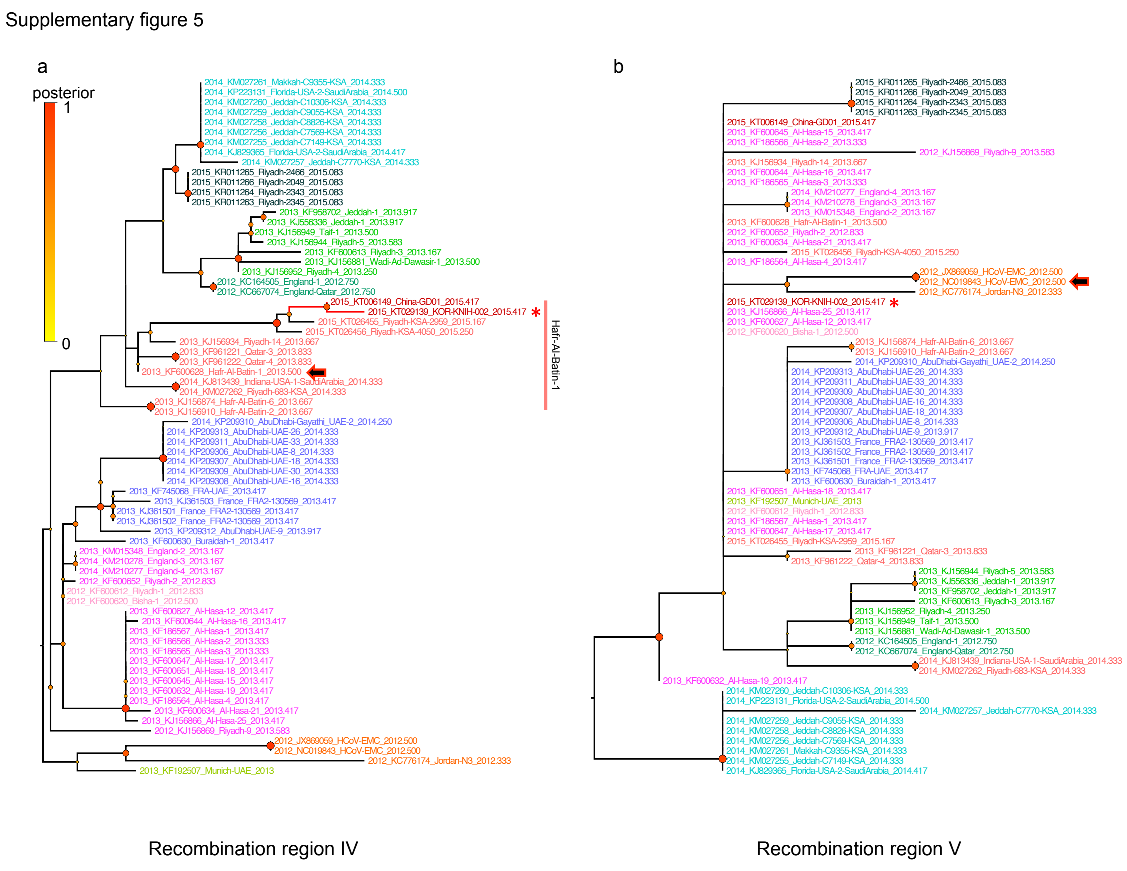

Supplement: Supplementary Information [file srep18825-s1.pdf]
